# Associations of phase angle and its change with all-cause mortality among community-dwelling older Japanese adults

**DOI:** 10.1038/s41598-026-35266-2

**Published:** 2026-01-18

**Authors:** Ei Teshima, Takanori Honda, Yu Setoyama, Satoko Sakata, Emi Oishi, Yoshihiko Furuta, Jun Hata, Yasuharu Nakashima, Toshiharu Ninomiya

**Affiliations:** 1 Department of Epidemiology and Public Health, Graduate School of Medical Sciences, Kyushu University, 3-1-1 Maidashi, Higashi-ku, Fukuoka, 812-8582 Japan; 2 Department of Orthopaedic Surgery, Graduate School of Medical Sciences, Kyushu University, Fukuoka, Japan; 3 Center for Cohort Studies, Graduate School of Medical Sciences, Kyushu University, Fukuoka, Japan; 4 Department of Medicine and Clinical Science, Graduate School of Medical Sciences, Kyushu University, Fukuoka, Japan; 5 Department of Health Care Administration and Management, Graduate School of Medical Sciences, Kyushu University, Fukuoka, Japan

**Keywords:** Bioelectrical impedance analysis, Phase angle, Mortality, Cohort study, Diseases, Health care, Medical research, Risk factors

## Abstract

Phase angle measured by bioimpedance is considered to reflect muscle quality and nutritional status. This study investigated the associations of phase angle and its 5-year change with all-cause mortality among community-dwelling older Japanese adults. A total of 1,291 Japanese adults aged ≥65 years with phase angle measured in 2012 were followed-up for a median of 10.3 years. Participants were divided into age-and sex-specific quartiles of phase angle and into quartiles of 5-year change in phase angle beyond standard age-related decline, defined as the difference between actually measured and estimated values from the linear regression line between phase angle values measured in 2007 and 2012. Hazard ratios (HRs) for all-cause mortality were estimated using Cox proportional hazards models. During follow-up, 347 participants died. The lowest quartile of phase angle showed a higher risk of all-cause mortality compared with the highest quartile after multivariable adjustment (HR 1.49, 95% CI 1.10–2.02). A similar trend was observed for 5-year change in phase angle beyond age-related decline (HR 1.42, 95% CI 1.03–1.97). Lower phase angle and greater decline beyond age-related expectations were associated with increased mortality risk. Phase angle may provide a simple, noninvasive measure of mortality risk in older adults.

**Supplementary Information:**

The online version contains supplementary material available at 10.1038/s41598-026-35266-2.

## Introduction

In older adults, age-related changes in body composition, such as loss of muscle mass, and malnutrition are significant risk factors for mortality and hospitalization^1–3^. To prevent early death and maintain independence, assessment of these physical conditions is beneficial.

Phase angle, calculated from the reactance and resistance of body tissues measured at 50 kHz using bioelectrical impedance analysis (BIA), reflects cell membrane condition and cellular integrity and is therefore considered an indicator of muscle quality and nutritional status^4–7^. A lower phase angle indicates reduced body cell mass or impaired cell membrane integrity. A relative decrease in phase angle is associated with several pathophysiological conditions, including decreased muscle mass and malnutrition^8,9^, which lead to a reduction in body cell mass, as well as chronic inflammation^9–11^, which may cause damage to cell membranes and an increase in extracellular fluid, all of which are closely related to frailty, functional decline, and increased vulnerability to adverse health outcomes. Strong associations between lower phase angle and mortality risk have been demonstrated in clinical settings, including among patients with kidney disease, cancer, cardiovascular disease, and critical illness^12–18^. However, few studies have examined this association in general populations^19–21^, and none have examined it in Asian general populations. Furthermore, most existing research has evaluated phase angle at a single time point; only one study has reported that a progressive decline in phase angle was associated with mortality risk^21^. Because changes in phase angle may reflect reduced muscle function, poor nutritional status, and systemic inflammation in addition to age-related decline, evaluating changes beyond standard age-related decline may provide valuable insights into mortality risk. Nonetheless, this issue has not been fully examined.

The present study aimed to investigate the associations between phase angle and all-cause mortality in a prospective cohort of community-dwelling older Japanese adults. In addition, we examined the association between 5-year changes in phase angle beyond standard age-related decline and mortality risk. This study may help to clarify the longitudinal impact of phase angle on health, and may help in screening older adults at higher risk of mortality.

## Methods

### Study population

The Hisayama Study is an ongoing community-based cohort study being conducted in the town of Hisayama, Fukuoka Prefecture, Japan. Details of the survey were reported previously^22^. In this study, annual health check-ups have been conducted among the residents aged 40 years and older since 1961. In addition, the geriatric health surveys assessing cognitive and physical function have been carried out every 5–7 years among the residents aged 65 years and older since 1985. For the present analysis, participants were selected according to the flowchart shown in Figure S1. In 2012, a total of 1,906 residents aged 65 years and older participated in the geriatric health surveys. For the present analysis of the association between phase angle levels and all-cause mortality, 615 of these participants were excluded: 44 participants who did not consent to enroll in this study, 545 without available data on phase angle in the 2012 survey, and 26 with phase angle outlier values in the 2012 survey (defined as values below the 1st percentile or above the 99th percentile), which were excluded to minimize the potential impact of measurement error. The remaining 1,291 participants were included. Among them, 1,220 had also participated in the 2007 health survey. After excluding 20 participants without 2007 phase angle data and 24 whose 2007 phase angle values were outliers, which were also excluded to minimize the potential impact of measurement error and to avoid the skewing of regression estimates of 5-year changes in phase angle, the remaining 1,176 participants were included in the analysis of 5-year changes in phase angle from 2007 to 2012 and their associations with all-cause mortality.

This study was approved by the Kyushu University Institutional Review Board for Clinical Research (Approval No. 23061), and all methods of this study were performed in accordance with the Declaration of Helsinki and Ethical Guidelines for Medical and Biological Research Involving Human Subjects in Japan (https://www.mext.go.jp/content/20250325-mxt_life-000035486-01.pdf). Informed consent was obtained from all the participants.

### Measurement of phase angle

BIA was performed using a multi-frequency segmental bioimpedance analyzer (MC-190; Tanita Corporation, Japan) in both the 2007 and 2012 surveys. Participants were instructed to remove any metal objects from their body prior to measurement, stand barefoot on the electrode plates, grip the metal handles with both hands, and keep their arms relaxed and hanging naturally at their sides during the measurement. The phase angle was calculated from the resistance (R) and reactance (Xc) values obtained at a frequency of 50 kHz based on the BIA data collected in both the 2007 and 2012 surveys. BIA measurements used a standard hand-to-foot measurement pathway, in which electrical current was applied between the left palm and the left sole, reflecting whole-body values. The following equation was used to calculate the phase angle: phase angle (°) = arctan (Xc/R) × (180°/π).

### Follow-up survey

Participants at the survey in 2012 were prospectively followed up from the date of evaluation to November 30, 2022. As previously reported, their health status was checked annually by mail or telephone for individuals who did not receive the annual health check-ups or who had moved away from town^22^. In addition, a daily monitoring system was established by the research team, local physicians, and staff from the town’s Health Office to receive information on mortality. One participant was lost to the follow-up.

### Measurement of other risk factors

Information on covariates was collected from the 2012 survey.

#### Questionnaire-based assessments

Each participant completed self-administered questionnaires on smoking habits, alcohol intake, regular exercise, and medications for hypertension, diabetes mellitus, and hypercholesterolemia. Smoking habits and alcohol intake were categorized as current use or not. Regular exercise was defined as exercise, including recreational walking, at least three times a week during leisure time. A dietary survey was conducted using a semi-quantitative food frequency questionnaire on dietary intake to estimate total energy intake and protein intake. Physical activity and functional status were assessed using the modified Rankin Scale (mRS). Participants were categorized into two groups: no functional limitation (mRS scores 0–1) and functional limitation (mRS scores 2–5). Depressive symptoms were assessed using the Geriatric Depression Scale (GDS), and a score of ≥5 was used to indicate the presence of depressive symptoms.

#### Anthropometric and physical performance measurements

Height and weight were measured in light clothing without shoes, and body mass index (BMI) was calculated. Handgrip strength was measured using a digital grip dynamometer (T.K.K. 5401; Takei Scientific Instruments, Japan), following the instructions of trained staff. Maximum handgrip strength was measured twice for each hand, and the maximum of the four measurements was used. Maximum gait speed was measured twice over a 5-m course, and the faster of the two measurements was used.

#### Blood pressure and electrocardiography measurement

Blood pressure was measured three times using an automatic sphygmomanometer after at least 5 minutes of rest in a seated position, and the mean of the three measurements was calculated. Hypertension was defined as a blood pressure of 140/90 mmHg or higher or current use of antihypertensive medications^23^. Electrocardiographic (ECG) abnormalities were defined as left ventricular hypertrophy (Minnesota code, 3-1), ST depression (4-1, 2, 3), and atrial fibrillation or atrial flutter (8-3).

#### Blood sampling and laboratory measurements

Blood samples were collected from an antecubital vein in the morning (approximately 7:00–10:00 a.m.), after an overnight fast. Plasma glucose levels were measured by the hexokinase method. Diabetes was defined as fasting plasma glucose levels ≥126 mg/dL, 2-hour post-load or casual glucose levels ≥200 mg/dL, or current use of oral antidiabetic agents or insulin^24^. Serum total cholesterol level was measured enzymatically, and hypercholesterolemia was defined as serum total cholesterol ≥220 mg/dL or ongoing treatment with lipid-lowering agents. Serum albumin was measured by the bromocresol green method. Serum high-sensitivity C-reactive protein (hs-CRP) levels were measured using a modification of the Behring Latex-Enhanced CRP assay on a BN-100 Nephelometer (Behring Diagnostics, Westwood, MA, USA).

### Statistical analysis

As shown in Table S1, participants were categorized into four groups based on quartiles of phase angle for each 5-year age group and sex stratum to minimize confounding by sex and age, because phase angle values decreased with age and were lower in women than in men. Trends in mean values and frequencies of risk factors across the age- and sex-specific phase angle levels were tested using linear regression analysis for continuous variables and logistic regression analysis for binary variables. Spearman’s rank correlation coefficients were calculated to examine the correlations between phase angle and handgrip strength, gait speed, serum hs-CRP, serum albumin, total energy intake, and protein intake.

All-cause mortality rates were calculated using the person-years method. Hazard ratios (HRs) and 95% confidence intervals (CIs) for all-cause mortality risk according to the age- and sex-specific phase angle levels were estimated using a Cox proportional hazards model. In multivariable analyses, the following variables were included as covariates in the relevant models: age, sex, hypertension, diabetes mellitus, hypercholesterolemia, BMI, ECG abnormalities, smoking habits, alcohol intake, and regular exercise. The covariates included in the multivariable models were selected based on previous studies examining associations with phase angle and mortality^9,12,19–21^, as well as on known risk factors for mortality. Additional models were constructed by separately adding the following sets of covariates to the multivariable model:

1. nutritional and inflammatory indicators, including serum albumin, total energy intake, protein intake, and log-transformed serum high-sensitivity C-reactive protein (hs-CRP);
2. physical function measures, including handgrip strength and gait speed; and
3. functional limitation and depressive symptoms, assessed by the modified Rankin Scale (mRS) and the Geriatric Depression Scale (GDS), respectively.
4. In sensitivity analyses, participants who died within the first year or the first two years of follow-up were excluded to account for potential reverse causation. Subgroup analyses were performed for age (< 75 or ≥ 75 years), sex, hypertension, diabetes mellitus, hypercholesterolemia, BMI (< 25, or ≥ 25 kg/m^2^), ECG abnormalities, smoking habits, alcohol intake, and regular exercise. In each subgroup, we compared mortality risk for participants in the first quartile of age- and sex-specific phase angle with that in the combined second to fourth quartiles. Heterogeneity between subgroups was tested by adding multiplicative interaction terms to the relevant Cox model. In the subsequent analyses, phase angle was analyzed without stratification by sex or age. The shape of the association between phase angle in 2012 and all-cause mortality risk was assessed by a multivariable-adjusted Cox model with restricted cubic splines, with five knots placed at the 5th, 27.5th, 50th, 72.5th, and 95th percentiles (4.17, 4.78, 5.20, 5.61, and 6.45 degrees, respectively) of phase angle in 2012, and the reference point set at the 50th percentile (5.20 degrees)^25^. In addition, we examined the association between 5-year changes in phase angle from the 2007 to 2012 surveys and all-cause mortality. For this analysis, a linear regression line was derived from phase angle data in 2007 and in 2012. The expected phase angle value in 2012 was estimated from this regression line, and the difference between the estimated and observed 2012 phase angle values was calculated as follows: [5-year change in phase angle] = [measured phase angle value in 2012] − [estimated phase angle in 2012]. This difference was defined as the 5-year change in phase angle beyond standard age-related decline. Participants were divided into quartiles of this change, and baseline characteristics and all-cause mortality risk were analyzed using the same methods as described above, with phase angle in 2007 added as a covariate. All statistical analyses were performed using SAS software (version 9.4; SAS Institute, Cary, NC, USA). A two-tailed *p* < 0.05 was considered statistically significant.

## Results

### Baseline characteristics of the study population

**Fig. 1:**
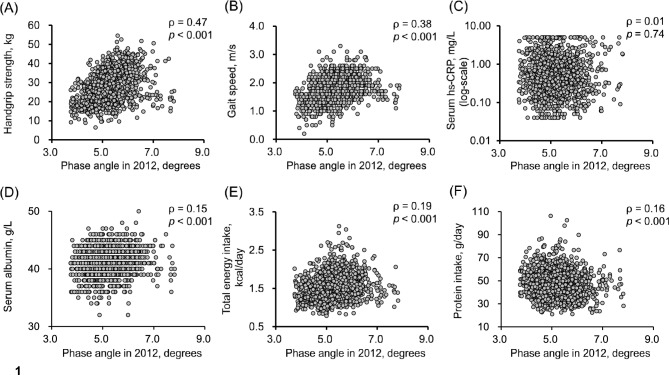
Correlation between phase angle in 2012 and handgrip strength (**A**), gait speed (**B**), serum high-sensitivity C-reactive protein (**C**), serum albumin (**D**), total energy intake (**E**), and protein intake (**F**). Abbreviations: Serum hs-CRP, serum high-sensitivity C-reactive protein. ρ indicates Spearman’s rank correlation coefficient.

Table 1 shows the baseline characteristics of study participants according to the age- and sex-specific quartiles of phase angle. The prevalence of hypertension and diabetes mellitus significantly increased with lower phase angle quartiles. Conversely, lower phase angle quartiles were significantly associated with lower frequencies of current alcohol intake as well as lower mean values of BMI, serum albumin, total energy intake, and muscle-function parameters, including handgrip strength and gait speed. Figure 1 shows scatterplots illustrating the correlations between phase angle and handgrip strength, gait speed, serum hs-CRP, serum albumin, total energy intake, and protein intake. Phase angle was significantly and positively correlated with handgrip strength (Spearman’s rank correlation coefficient: ρ = 0.47, *p* < 0.001) and gait speed (ρ = 0.38, *p* < 0.001). Significant but weak correlations were observed between phase angle value and serum albumin, total energy intake, and protein intake (ρ = 0.15, 0.19 and 0.16, respectively). Phase angle was not correlated with serum hs-CRP (ρ = 0.01, *p* = 0.74).

**Table 1:** Baseline characteristics of participants in the 2012 survey according to age- and sex-specific quartiles of phase angle measured in 2012.

Variables	Total population (n = 1,291)	Quartiles of phase angle				*p* for trend
		Q1 (n = 319)	Q2 (n = 324)	Q3 (n = 327)	Q4 (n = 321)	
Phase angle, degrees	5.2 (0.7)	4.6 (0.4)	5.0 (0.4)	5.4 (0.4)	6.0 (0.6)	< 0.001
Age, years	74.2 (6.4)	74.3 (6.6)	74.1 (6.4)	74.1 (6.4)	74.1 (6.4)	0.58
Women, %	56.3	56.4	56.2	56.3	56.4	1.00
Hypertension, %	70.7	75.2	72.5	67.6	67.6	0.01
Diabetes mellitus, % ^a)^	24.4	26.4	30.2	23.1	18.0	0.003
Hypercholesterolemia, %	54.8	52.7	55.9	52.9	57.6	0.34
Body mass index, kg/m^2^	23.2 (3.3)	22.5 (3.5)	23.0 (3.4)	23.5 (3.2)	23.6 (3.1)	< 0.001
ECG abnormalities, % ^b)^	16.5	17.0	14.8	16.5	17.8	0.66
Smoking habits, %	8.2	9.7	9.3	6.7	7.2	0.14
Alcohol intake, %	41.4	36.4	40.7	44.7	43.6	0.04
Regular exercise, %	40.6	36.7	41.1	41.3	43.3	0.10
Serum albumin, g/L ^c)^	41.2 (2.4)	40.7 (2.4)	41.2 (2.4)	41.3 (2.3)	41.6 (2.3)	< 0.001
Total energy intake, kcal/day ^d)^	1,531.0 (331.7)	1,484.3 (309.4)	1,541.1 (342.4)	1,554.1 (330.6)	1,543.6 (340.4)	0.02
Protein intake, g/day ^d)^	47.4 (11.6)	45.5 (11.1)	47.3 (11.5)	48.2 (11.4)	48.5 (12.2)	< 0.001
Serum hs-CRP, mg/L ^e)^	0.46 (0.24–1.04)	0.54 (0.27–1.15)	0.43 (0.19–0.98)	0.44 (0.24–0.94)	0.45 (0.24–1.04)	0.12
Functional limitation, %	17.2	25.7	15.4	12.5	15.3	< 0.001
Depressive symptoms, % ^f)^	21.6	28.9	21.1	17.8	18.9	0.002
*Muscle function parameters*						
Handgrip strength, kg ^e)^	26.8 (8.2)	24.8 (7.6)	26.7 (8.2)	28.0 (8.0)	27.7 (8.7)	< 0.001
Gait speed, m/s ^g)^	1.8 (0.4)	1.7 (0.5)	1.7 (0.4)	1.8 (0.4)	1.8 (0.4)	< 0.001

Abbreviations: ECG, electrocardiogram; hs-CRP, high-sensitivity C-reactive protein.

Data are shown as means (standard deviations) for continuous variables, except for serum hs-CRP, which is shown as median (interquartile range), and as percentages for categorical variables.

a) Missing in 26 participants.

b) Missing in 1 participant.

c) Missing in 2 participants.

d) Missing in 9 participants.

e) Missing in 3 participants.

f) Missing in 33 participants.

g) Missing in 136 participants.

### Phase angle and all-cause mortality

**Fig. 2:**
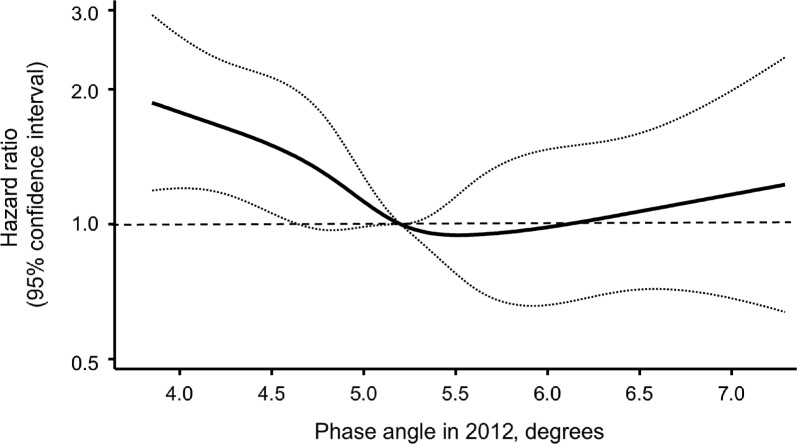
Association between phase angle in 2012 and all-cause mortality risk during a median 10.3-year follow-up, as assessed using a multivariable-adjusted Cox proportional hazards model with restricted cubic splines. The solid line and dashed lines represent the estimated hazard ratio and its 95% confidence intervals, respectively. Knots were placed at the 5th, 27.5th, 50th, 72.5th, and 95th percentiles (4.17, 4.80, 5.20, 5.61, and 6.45 degrees, respectively) of phase angle in 2012. The reference point was set at the 50th percentile (5.20 degrees) of phase angle in 2012. The X-axis represents values of phase angle in 2012, ranging from the 1st percentile (3.84 degrees) to the 99th percentile (7.29 degrees). The risk estimates were adjusted for baseline covariates, including age, sex, hypertension, diabetes mellitus, hypercholesterolemia, body mass index, electrocardiographic abnormalities, smoking habits, alcohol intake, and regular exercise.

During a median follow-up of 10.3 years, 347 participants (215 men and 132 women) died from any cause. Table 2 shows the estimated all-cause mortality risk according to the age- and sex-specific quartiles of phase angle. The age- and sex-adjusted HR for all-cause mortality was significantly higher in the lowest quartile compared with the highest (HR 1.70, 95% CI 1.27–2.27) (Table 2, Model 1). This association remained significant after adjustment for potential confounding factors (HR 1.49, 95% CI 1.10–2.02) (Table 2, Model 2). When additional adjustments were made for serum albumin, total energy intake, protein intake, and serum hs-CRP, the associations were slightly attenuated but remained statistically significant (Table 2, Model 3). In addition, further adjustment for functional limitation and depressive symptoms, similar association was observed. However, further adjustment for muscle-function parameters, including handgrip strength and gait speed, attenuated the associations to a non-significant level (Table S2). In sensitivity analyses excluding participants who died within the first year and within the first two years of follow-up period, the results were substantially unchanged with the main analysis, although statistical significance was slightly attenuated in the two-year exclusion analysis (Table S3). In subgroup analyses stratified by age, sex, hypertension, diabetes mellitus, hypercholesterolemia, BMI, ECG abnormalities, smoking habits, alcohol intake, and regular exercise, no heterogeneity was observed (*p* for heterogeneity ≥ 0.10), except for ECG abnormalities (*p* for heterogeneity = 0.01) (Table S4). We also assessed the shape of the association between phase angle in 2012 and all-cause mortality using a multivariable-adjusted Cox model with restricted cubic splines. The risk of all-cause mortality increased with decreasing phase angle below the median level used as the reference, such that the lower limit of the 95% CI for the HR exceeded 1.0 at a phase angle of 4.6 degrees (Figure 2).

**Table 2:** Hazard ratios and 95% confidence intervals for all-cause mortality according to age- and sex-specific quartiles of phase angle during a median 10.3-year follow-up (2012–2022).

Phase angle quartile in 2012	N of events /participants	Crude mortality rate, per 10^3^ PYs	Model 1^a)^		Model 2^b)^		Model 3^c)^	
			Hazard ratio (95% CI)	*p* value	Hazard ratio (95% CI)	*p* value	Hazard ratio (95% CI)	*p* value
Q1	110/319	38.4	1.70 (1.27–2.27)	< 0.001	1.49 (1.10–2.02)	0.01	1.37 (1.003–1.87)	0.048
Q2	84/324	28.1	1.16 (0.85–1.58)	0.35	1.03 (0.75–1.42)	0.84	1.01 (0.73–1.40)	0.97
Q3	75/327	24.2	1.01 (0.74–1.39)	0.94	0.95 (0.69–1.32)	0.77	0.93 (0.67–1.29)	0.66
Q4	78/321	25.4	1.00 (reference)		1.00 (reference)		1.00 (reference)	
*p* for trend				< 0.001		0.009		0.04

Abbreviations: PYs, person-years; CI, confidence interval.

a) Model 1: Adjusted for age and sex

b) Model 2: Adjusted for age, sex, hypertension, diabetes mellitus, hypercholesterolemia, body mass index, electrocardiogram abnormalities, smoking habits, alcohol intake, and regular exercise.

c) Model 3: Adjusted for covariates included in model 2 plus serum albumin, total energy intake, protein intake and serum high-sensitivity C-reactive protein (log-transformed) as nutritional and inflammatory indicators.

### 5-year change in phase angle and all-cause mortality

**Fig. 3:**
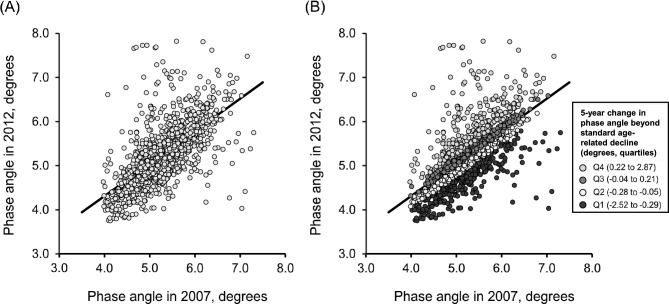
Association between phase angle in 2007 and phase angle in 2012. **(A)** Scatterplot showing the association between phase angle measured in 2007 and 2012, with the fitted linear regression line ([phase angle in 2012] = 0.74 × [phase angle in 2012 in 2007] + 1.36). **(B)** Scatterplot showing the distribution of individual values color-coded according to quartiles of 5-year change in phase angle beyond standard age-related decline.

Figure 3 shows a scatterplot of phase angle in 2007 and 2012, along with the regression line from the linear regression analysis. Because phase angle values declined with aging, we assumed that the linear regression line derived from the 2007 and 2012 phase angle data represented the standard age-related decline in this population. Based on this line, we estimated the expected phase angle in 2012 and calculated the difference between this estimated and the actual measured phase angle. This difference was defined as the 5-year change in phase angle beyond standard age-related decline, meaning that a more negative value reflected a greater reduction in phase angle than expected from normal aging in this population.

Table S5 shows the baseline characteristics of participants by quartiles of 5-year change in phase angle beyond standard age-related decline. Participants with a greater 5-year decline in phase angle beyond standard age-related decline were older and more likely to be women. A greater decline in phase angle was also associated with a higher prevalence of hypertension and diabetes mellitus, lower mean values of BMI, serum albumin, total energy intake, handgrip strength, and gait speed, and with lower frequencies of current alcohol intake and regular exercise. Regarding all-cause mortality, participants in the lowest quartile of 5-year change in phase angle beyond standard age-related decline had a significantly higher multivariable-adjusted HR compared with those in the highest quartile (HR 1.42, 95% CI 1.03–1.97) (Table 3, Model 2). However, this association was attenuated to a non-significant level after additional adjustment for serum albumin, total energy intake, protein intake and serum hs-CRP (Table 3, Model 3).

**Table 3:** Hazard ratios and 95% confidence intervals for all-cause mortality according to quartiles of 5-year change in phase angle beyond standard age-related decline during a median 10.3-year follow-up (2012–2022).

5-year change in phase angle (quartile, degrees)	N of events /participants	Crude mortality rate, per 10^3^ PYs	Model 1^a)^		Model 2^b)^		Model 3^c)^	
			Hazard ratio (95% CI)	*p* value	Hazard ratio (95% CI)	*p* value	Hazard ratio (95% CI)	*p* value
Q1 (-2.52 to -0.29)	121/294	48.0	1.64 (1.19–2.25)	0.002	1.42 (1.03–1.97)	0.03	1.28 (0.92–1.79)	0.15
Q2 (-0.28 to -0.05)	66/294	23.6	1.00 (0.70–1.42)	0.99	0.90 (0.63–1.29)	0.58	0.86 (0.60–1.23)	0.40
Q3 (-0.04 to 0.21)	61/294	21.6	0.89 (0.62–1.26)	0.50	0.89 (0.62–1.28)	0.53	0.88 (0.62–1.27)	0.50
Q4 (0.22 to 2.87)	64/294	22.4	1.00 (reference)		1.00 (reference)		1.00 (reference)	
*p* for trend				< 0.001		0.02		0.11

Abbreviations: PYs, person-years; CI, confidence interval.

a) Model 1: Adjusted for age, sex, and phase angle in 2007.

b) Model 2: Adjusted for age, sex, phase angle in 2007, hypertension, diabetes mellitus, hypercholesterolemia, body mass index, electrocardiogram abnormalities, smoking habits, alcohol intake, and regular exercise.

c) Model 3: Adjusted for covariates included in model 2 plus serum albumin, total energy intake, protein intake and serum high-sensitivity C-reactive protein (log-transformed) as nutritional and inflammatory indicators.

## Discussion

In this 10-year follow-up study of community-dwelling older Japanese adults, lower phase angle was significantly associated with higher risk of all-cause mortality, and this association persisted after multivariable adjustment for potential confounders, including chronic diseases, BMI, and lifestyle factors. Furthermore, a greater 5-year decline in phase angle beyond the standard age-related decline was significantly associated with higher of all-cause mortality. Our findings suggest that phase angle and its change may serve as surrogate indicators of muscle strength and physical performance, and may provide a simple, non-invasive means of predicting mortality in older Japanese adults.

Several clinical studies have reported an association between phase angle and mortality in clinical settings^12–18^. A recent systematic review showed that lower phase angle was significantly associated with higher mortality risk in studies of patients receiving hemodialysis, and in those with sepsis, advanced cancer, liver cirrhosis, chronic obstructive pulmonary disease, or intensive care unit admission^12^. A community-based study of U.S. residents aged 60 years or older demonstrated that lower phase angle was significantly associated with higher mortality risk^19^. Our findings in community-dwelling older Japanese adults are consistent with these previous observations. In addition, the present study provides additional insight into the potential mechanisms underlying this association. In our analyses, the inverse association between phase angle and all-cause mortality was attenuated but remained significant after adjustment for indicators of nutritional status, inflammation, functional limitations, and depressive symptoms. This indicates that phase angle reflects overall health that are not fully explained by these conventional risk factors alone. In contrast, additional adjustment for muscle-function parameters, which were strongly correlated with phase angle, further attenuated the association to a non-significant level. This suggests that phase angle largely reflects muscle function, and that declining muscle function may contribute to the excess mortality risk. Phase angle is influenced by factors such as cellular mass, cell membrane integrity, and the intracellular/extracellular water ratio^7,10^. Reflecting these physiological properties, phase angle has also been shown to be associated with muscle quantity and quality^5–7^, and that exercise interventions can improve phase angle values^26–28^. Accordingly, lower phase angle values may indicate impaired muscle function, which represents a key feature of sarcopenia and frailty in older adults. Declines in muscle function may reflect cumulative effects of aging, malnutrition, chronic inflammation, and low physical activity. The partial attenuation observed after adjustment for nutritional and inflammatory indicators supports the view that these factors contribute to lower phase angle values, and the associated excess mortality risk. The present results suggest that the association between phase angle and all-cause mortality in community-dwelling older adults is largely explained by its close relationship with muscle function, while also reflecting broader aspects of overall health, including nutritional status and inflammatory burden. Phase angle may therefore provide a simple, noninvasive measure of age-related functional decline in public health settings.

In the present study, there was no heterogeneity in the association between phase angle and mortality across most subgroups, except for those with ECG abnormalities. Participants with ECG abnormalities tended to show a stronger association between lower phase angle and higher mortality risk than those without. The exact reason for the heterogeneity observed in this subgroup remains unclear, and the finding should be interpreted with caution. Although this finding may be due to chance, it is also possible that ECG abnormalities reflect accumulated cardiovascular risk factors, which could have contributed to the stronger association observed.

To our knowledge, only one study has reported an association between changes in phase angle and mortality. That study, conducted in a healthy adult Danish population, found that a decline in phase angle over 6 years was significantly associated with an increased risk of mortality over the following 18 years^21^. These results are consistent with our findings, suggesting that the prognostic significance of phase angle decline beyond age-related changes may be consistent across different racial and ethnic populations. However, that study assessed phase angle change as a simple absolute difference, which may have been influenced by age-related decline. Because phase angle declines with age, the simple change in phase angle used in the previous study may have been strongly influenced by aging itself. Therefore, in the present study, we attempted to minimize the influence of age-related decline on changes in phase angle. Specifically, we estimated the standard 5-year age-related change in phase angle in this population using the regression line derived from measurements taken in 2007 and 2012 and then evaluated whether each participant’s actual decline was greater or smaller than the expected value, based on the difference between the measured and estimated phase angles. As a result, a greater decline in phase angle than the standard age-related decline was significantly associated with an increased risk of all-cause mortality. Our findings extend this previous work by demonstrating that a decline in phase angle exceeding the expected age-related change is associated with an increased risk of all-cause mortality, suggesting that accelerated deterioration in cellular integrity and body composition beyond normal aging may have important prognostic implications.

On the other hand, this association was attenuated to a non-significant level after adjustment for indicators of nutritional status and inflammation. Several studies have suggested that poor nutritional status and inflammatory markers are associated with decreased phase angle^8–11^. Therefore, these findings suggest that nutritional and inflammatory factors may accelerate the decline in phase angle and, in turn, contribute to the increased risk of mortality.

The strengths of the present study include the high participation rate in screening tests at baseline, an almost complete follow-up rate (99.9%), and the examination of changes in phase angle and mortality using residual values to strictly control for confounding by age. Several limitations of our study should also be noted. First, there is a possibility of survivor bias because our analysis included only participants who survived until the 2012 survey and had phase angle measurements. The 121 participants who died after the 2007 survey and before 2012 survey were older and had lower phase angle values at the 2007 survey compared with those included in the present analysis, possibly leading to an underestimation of the association between the change of the phase angle and mortality risk. Second, participants who were unable to undergo BIA measurement—such as those who could not stand unassisted, had pacemakers, or were unable to attend the examination—were excluded from the analysis. This could have led to selection bias, resulting in participants being healthier than the general population. Because the excluded participants tended to be older, were more often female, and had more comorbidities than those who could be measured, their phase angles were likely lower and their 10-year mortality rate higher. Therefore, the all-cause mortality observed in the current study may have been underestimated. Third, phase angle in 2012 was measured only once, and 5-year change in phase angle was derived from only two time points (2007 and 2012), which might not fully capture short-term fluctuations or nonlinear trajectories in phase angle over time. Fourth, although we adjusted for multiple potential confounders, residual confounding by unmeasured variables such as socioeconomic factors cannot be ruled out. Finally, the generalizability of the present findings may be limited because the study was conducted in only a single region of Japan. Therefore, further investigations in different populations and settings are warranted.

In conclusion, lower phase angle was associated with higher all-cause mortality in a community-dwelling older Japanese population. We also found that a greater 5-year decline in phase angle beyond the standard age-related decline was associated with excess mortality risk. Our findings that phase angle value and its change may reflect risk of early death due to functional decline underscore the potential usefulness of incorporating phase angle measurement using noninvasive, feasible BIA assessment into routine clinical and public health practice.

## Supplementary Information

Supplementary Information.

## Data Availability

The datasets generated and analyzed in the present study are not publicly available because they contain confidential clinical and demographic data of the study participants. However, further information about the datasets is available with the permission of the principal investigator of the Hisayama Study (Toshiharu Ninomiya) on reasonable request for purposes of replicating procedures and results.
