# Supplementary material for: Associations of phase angle and its change with all-cause mortality among community-dwelling older Japanese adults
Source: Sci Rep. 2026 Jan 18;16:5539. doi: 10.1038/s41598-026-35266-2 (PMC12886904; doi:10.1038/s41598-026-35266-2)
Supplement: Supplementary file 1 — Supplementary Information. [file 41598_2026_35266_MOESM1_ESM.docx]

| **Table S1.** Ranges of phase angle in 2012 according to age- and sex-specific quartiles | | | | | |
| --- | --- | --- | --- | --- | --- |
| Age, years | Mean value of  phase angle in 2012, degrees | Quartiles of phase angle in 2012, degrees | | | |
|  |  | Q1 | Q2 | Q3 | Q4 |
| **Men** |  |  |  |  |  |
| 65–69 | 5.771 | < 5.403 | 5.403–5.745 | 5.746–6.083 | > 6.083 |
| 70–74 | 5.721 | < 5.344 | 5.344–5.652 | 5.653–6.057 | > 6.057 |
| 75–79 | 5.412 | < 4.990 | 4.990–5.438 | 5.439–5.865 | > 5.865 |
| 80–84 | 5.004 | < 4.714 | 4.714–5.019 | 5.020–5.349 | > 5.349 |
| 85– | 4.822 | < 4.528 | 4.528–4.782 | 4.783–5.176 | > 5.176 |
| **Women** |  |  |  |  |  |
| 65–69 | 5.257 | < 4.810 | 4.810–5.162 | 5.163–5.552 | > 5.552 |
| 70–74 | 5.152 | < 4.798 | 4.798–5.091 | 5.092–5.443 | > 5.443 |
| 75–79 | 4.982 | < 4.599 | 4.599–4.872 | 4.873–5.308 | > 5.308 |
| 80–84 | 4.778 | < 4.329 | 4.329–4.651 | 4.652–5.086 | > 5.086 |
| 85– | 4.582 | < 4.172 | 4.172–4.329 | 4.330–4.807 | > 4.807 |

| **Table S2.** Hazard ratios and 95% confidence intervals for all-cause mortality according to quartiles of phase angle in 2012 after additional adjustment for muscle function parameters and functional limitation and depressive symptoms during a median 10.3-year follow-up (2012–2022) | | | | |
| --- | --- | --- | --- | --- |
| Phase angle quartile  in 2012 | N of events/participants | Crude mortality rate,  per 10^3^ PYs | Hazard ratio (95% CI) | *p* value |
| ***Multivariable-adjusted HR including muscle-function parameters ^a)^*** | | | | |
| Q1 | 89/260 | 37.9 | 1.21 (0.86–1.70) | 0.28 |
| Q2 | 68/282 | 25.9 | 0.95 (0.67–1.34) | 0.75 |
| Q3 | 61/296 | 21.6 | 0.91 (0.64–1.29) | 0.58 |
| Q4 | 66/291 | 23.6 | 1.00 (reference) |  |
| *p* for trend |  |  |  | 0.26 |
| ***Multivariable-adjusted HR including functional limitation and depressive symptoms ^b)^*** | | | | |
| Q1 | 98/296 | 36.6 | 1.39 (1.02–1.91) | 0.04 |
| Q2 | 78/307 | 27.5 | 1.06 (0.76–1.47) | 0.73 |
| Q3 | 70/314 | 23.4 | 0.95 (0.69–1.32) | 0.77 |
| Q4 | 74/313 | 24.7 | 1.00 (reference) |  |
| p for trend |  |  |  | 0.03 |
| Abbreviations: PYs, person-years; CI, confidence interval. | | | | |
| 1. Adjusted for age, sex, hypertension, diabetes mellitus, hypercholesterolemia, body mass index, electrocardiogram abnormalities, smoking habits, alcohol intake, regular exercise (Model2 in Table 2), and muscle-function parameters—namely, handgrip strength and gait speed. 2. Adjusted for age, sex, hypertension, diabetes mellitus, hypercholesterolemia, body mass index, electrocardiogram abnormalities, smoking habits, alcohol intake, regular exercise (Model2 in Table 2), and functional limitation and depressive symptoms. | | | | |

| **Table S3.** Sensitivity analysis of the association between age- and sex-specific quartiles of phase angle in 2012 and all-cause mortality during a median 10.3-year follow- up (2012–2022), excluding participants who died within the first year of follow-up | | | | | | | | | | | |
| --- | --- | --- | --- | --- | --- | --- | --- | --- | --- | --- | --- |
| Phase angle  quartile in 2012 | N of events/ participants | Crude mortality rate,  per 10^3^ PYs | Model 1 ^a)^ | |  | Model 2 ^b)^ | |  | Model 3 ^c)^ | |  |
|  |  |  | Hazard ratio  (95% CI) | *p* value |  | Hazard ratio  (95% CI) | *p* value |  | Hazard ratio  (95% CI) | *p* value |  |
| Excluding deaths within 1 year | | | | | | | | | | |  |
| Q1 | 105/314 | 36.7 | 1.63 (1.22–2.19) | 0.001 |  | 1.43 (1.05–1.95) | 0.02 |  | 1.32 (0.96–1.81) | 0.08 |  |
| Q2 | 81/321 | 27.1 | 1.12 (0.82–1.53) | 0.47 |  | 1.00 (0.73–1.39) | 0.99 |  | 0.98 (0.70–1.36) | 0.88 |  |
| Q3 | 72/324 | 23.3 | 0.97 (0.71–1.34) | 0.86 |  | 0.92 (0.66–1.27) | 0.60 |  | 0.89 (0.64–1.24) | 0.50 |  |
| Q4 | 78/321 | 25.4 | 1.00 (reference) |  |  | 1.00 (reference) |  |  | 1.00 (reference) |  |  |
| *p* for trend |  |  |  | < 0.001 |  |  | 0.02 |  |  | 0.06 |  |
| Excluding deaths within 2 years | | | | | | | | | | |  |
| Q1 | 99/308 | 34.53 | 1.60 (1.19–2.16) | 0.002 |  | 1.37 (0.998–1.88) | 0.051 |  | 1.26 (0.91–1.73) | 0.17 |  |
| Q2 | 73/313 | 24.45 | 1.04 (0.75–1.43) | 0.81 |  | 0.92 (0.66–1.29) | 0.63 |  | 0.89(0.64–1.26) | 0.52 |  |
| Q3 | 68/320 | 21.98 | 0.95 (0.68–1.31) | 0.74 |  | 0.89 (0.64–1.24) | 0.51 |  | 0.87 (0.62–1.22) | 0.41 |  |
| Q4 | 76/319 | 24.78 | 1.00 (reference) |  |  | 1.00 (reference) |  |  | 1.00 (reference) |  |  |
| *p* for trend |  |  |  | 0.002 |  |  | 0.049 |  |  | 0.16 |  |
| Abbreviations: PYs, person-years; CI, confidence interval.   1. Model 1: Adjusted for age and sex. 2. Model 2: Adjusted for age, sex, hypertension, diabetes mellitus, hypercholesterolemia, body mass index, electrocardiogram abnormalities, smoking habits, alcohol intake, and regular exercise. 3. Model 3: Adjusted for covariates included in Model 2 plus serum albumin, total energy intake, protein intake, and serum high-sensitivity C-reactive protein (log-transformed). | | | | | | | | | | | |

| **Table S4.** Subgroup analysis of the association between age- and sex-specific quartiles of phase angle in 2012 and all-cause mortality during a median 10.3-year follow-up (2012–2022) | | | | | | |
| --- | --- | --- | --- | --- | --- | --- |
| Variables | Subgroup | N of events/participants at risk | | Hazard ratio (95% CI) in Q1 vs Q2–Q4 ^a)^ | *p* value | *p* for heterogeneity |
|  |  | Q1 | Q2–4 |  |  |  |
| ***Overall*** |  | 110/319 | 237/972 | 1.49 (1.18–1.89) | 0.001 |  |
| ***Age*** |  |  |  |  |  |  |
|  | < 75 years | 34/180 | 62/549 | 1.64 (1.06–2.53) | 0.03 | 0.58 |
|  | ≥ 75 years | 76/139 | 175/423 | 1.37 (1.03–1.82) | 0.03 |  |
| ***Sex*** |  |  |  |  |  |  |
|  | women | 39/180 | 93/547 | 1.28 (0.86–1.89) | 0.22 | 0.19 |
|  | men | 71/139 | 144/425 | 1.73 (1.28–2.35) | < 0.001 |  |
| ***Hypertension*** | |  |  |  |  |  |
|  | (-) | 18/79 | 50/299 | 1.32 (0.72–2.42) | 0.36 | 0.78 |
|  | (+) | 92/240 | 187/673 | 1.54 (1.18–2.00) | 0.001 |  |
| ***Diabetes mellitus*** | |  |  |  |  |  |
|  | (-) | 62/229 | 159/727 | 1.26 (0.93–1.70) | 0.14 | 0.10 |
|  | (+) | 43/82 | 71/227 | 2.04 (1.37–3.05) | < 0.001 |  |
| ***Hypercholesterolemia*** | |  |  |  |  |  |
|  | (-) | 66/151 | 133/433 | 1.63 (1.19–2.23) | 0.003 | 0.53 |
|  | (+) | 44/168 | 104/539 | 1.35 (0.94–1.94) | 0.11 |  |
| ***Body mass index*** | |  |  |  |  |  |
|  | < 25.0 kg/m^2^ | 89/254 | 174/704 | 1.51 (1.16–1.96) | 0.002 | 0.77 |
|  | ≥ 25.0 kg/m^2^ | 21/65 | 63/268 | 1.68 (0.99–2.84) | 0.053 |  |
| ***ECG abnormalities*** | |  |  |  |  |  |
|  | (-) | 82/264 | 191/813 | 1.28 (0.97–1.68) | 0.08 | 0.01 |
|  | (+) | 27/54 | 46/159 | 2.91 (1.76–4.82) | < 0.001 |  |
| ***Smoking habits*** | |  |  |  |  |  |
|  | (-) | 98/288 | 210/897 | 1.58 (1.23–2.03) | < 0.001 | 0.28 |
|  | (+) | 12/31 | 27/75 | 0.98 (0.46–2.06) | 0.95 |  |
| ***Alcohol intake*** | |  |  |  |  |  |
|  | (-) | 65/203 | 143/554 | 1.36 (1.00–1.86) | 0.05 | 0.46 |
|  | (+) | 45/116 | 94/418 | 1.79 (1.22–2.63) | 0.003 |  |
| ***Regular exercise*** | |  |  |  |  |  |
|  | (-) | 116/202 | 418/565 | 1.57 (1.17–2.10) | 0.003 | 0.67 |
|  | (+) | 34/117 | 97/407 | 1.34 (0.88–2.05) | 0.17 |  |
| Abbreviations: CI, confidence interval; ECG, electrocardiogram.  In this analysis, since no significant differences in all-cause mortality risk were observed among the Q2–Q4 groups and a significantly higher risk was observed in the Q1 group, we evaluated the all-cause mortality risk of the Q1 group in comparison with the combined Q2–Q4 groups. a) Adjusted for age, sex, hypertension, diabetes mellitus, hypercholesterolemia, body mass index, electrocardiogram abnormalities, smoking habits, alcohol intake, and regular exercise, where the variables used for subgroup analyses were excluded from the covariates. | | | | | | |

| **Table S5.** Baseline characteristics of participants in the 2012 survey according to quartiles of 5-year change in phase angle beyond standard age-related decline | | | | | | | |
| --- | --- | --- | --- | --- | --- | --- | --- |
| Variables | Total population  (n = 1,176) | Quartiles of 5-year change in phase angle  beyond standard age-related decline (degrees) | | | | *p* for trend |  |
|  |  | Q1  (-2.52 to -0.29)  (n = 294) | Q2  (-0.28 to -0.05)  (n = 294) | Q3  (-0.04 to 0.21)  (n = 294) | Q4  (0.22 to 2.87)  (n = 294) |  |  |
| 5-year change in phase angle, degrees | 0.00 (0.53) | -0.56 (0.29) | -0.16 (0.07) | 0.08 (0.08) | 0.64 (0.52) | < 0.001 |  |
| Phase angle in 2007, degrees | 5.29 (0.61) | 5.24 (0.66) | 5.20 (0.59) | 5.29 (0.57) | 5.40 (0.61) | < 0.001 |  |
| Phase angle in 2012, degrees | 5.26 (0.70) | 4.67 (0.46) | 5.04 (0.45) | 5.34 (0.44) | 5.98 (0.64) | < 0.001 |  |
|  |  |  |  |  |  |  |  |
| Age, years | 74.3 (6.4) | 77.2 (6.7) | 74.4 (6.3) | 72.8 (6.0) | 72.6 (5.5) | < 0.001 |  |
| Women, % | 56.1 | 62.2 | 62.6 | 52.4 | 47.3 | < 0.001 |  |
| Hypertension, % | 69.9 | 77.2 | 72.1 | 65.3 | 65.0 | < 0.001 |  |
| Diabetes mellitus, % ^a)^ | 24.7 | 30.9 | 25.9 | 19.0 | 23.1 | 0.009 |  |
| Hypercholesterolemia, % | 54.2 | 53.1 | 55.8 | 54.1 | 53.7 | 0.98 |  |
| Body mass index, kg/m^2^ | 23.2 (3.3) | 23.0 (3.6) | 23.1 (3.2) | 23.1 (3.1) | 23.5 (3.3) | 0.045 |  |
| ECG abnormalities, % | 16.2 | 16.0 | 17.4 | 17.7 | 14.0 | 0.55 |  |
| Smoking habits, % | 7.4 | 6.5 | 5.4 | 7.8 | 9.9 | 0.07 |  |
| Alcohol intake, % | 41.2 | 30.3 | 40.8 | 44.6 | 49.3 | < 0.001 |  |
| Regular exercise, % | 40.8 | 35.4 | 37.8 | 45.2 | 44.9 | 0.005 |  |
| Serum albumin, g/L ^b)^ | 41.2 (2.4) | 40.5 (2.4) | 41.1 (2.3) | 41.3 (2.3) | 41.7 (2.3) | < 0.001 |  |
| Total energy intake, kcal/day ^c)^ | 1528.3 (324.7) | 1499.7 (314.4) | 1517.9 (306.7) | 1524.6 (310.5) | 1570.6 (361.5) | 0.01 |  |
| Protein intake, g/day ^c)^ | 47.4 (11.5) | 45.8 (10.8) | 47.0 (10.4) | 47.6 (11.2) | 49.1 (13.2) | < 0.001 |  |
| Serum hs-CRP, mg/L ^d)^ | 0.46 (0.23–1.03) | 0.54 (0.24–1.23) | 0.47 (0.25–1.01) | 0.44 (0.23–0.92) | 0.42 (0.21–0.98) | 0.10 |  |
| Functional limitation, % | 16.8 | 31.0 | 12.2 | 10.9 | 12.9 | < 0.001 |  |
| Depressive symptoms, % ^e)^ | 20.7 | 26.8 | 20.0 | 19.2 | 16.7 | 0.004 |  |
| ***Muscle function parameters*** | |  |  |  |  |  |  |
| Handgrip strength, kg ^b)^ | 26.9 (8.2) | 23.9 (7.3) | 26.3 (7.7) | 27.9 (8.1) | 29.3 (8.5) | < 0.001 |  |
| Gait speed, m/s ^f)^ | 1.76 (0.42) | 1.53 (0.43) | 1.78 (0.37) | 1.86 (0.39) | 1.85 (0.39) | < 0.001 |  |
| Abbreviations: ECG, electrocardiogram; hs-CRP, high-sensitivity C-reactive protein.  Data are shown as means (standard deviations) for continuous variables, except for serum hs-CRP, which is shown as the median (interquartile range), and as percentages for categorical variables.   1. Missing in 25 participants. 2. Missing in 2 participants. 3. Missing in 6 participants. 4. Missing in 3 participants. 5. Missing in 29 participants. 6. Missing in 120 participants. | | | | | | | |

Participants underwent the examination in 2007 (n = 1,220)

Hisayama residents aged ≥65 years

underwent the geriatric health surveys in 2012 (n = 1,906)

(Excluded)

- Did not consent to participate in the present study (n = 44)
- Missing data on phase angle in 2012 (n = 545)
- Phase angle in 2012 below the 1st or above the 99th percentile (n = 26)

Examined participants for the association between phase angle in 2012 and mortality (n = 1,291)

(Excluded)

- Missing data on phase angle in 2007 (n = 20)
- Phase angle in 2007 below the 1st or above the 99th percentile (n = 24)

Examined participants for the association between changes in phase angle from 2007 to 2012 and mortality (n = 1,176)

**Figure S1.** Flowchart of the study population selection.
